# A novel sub-epidemic modeling framework for short-term forecasting epidemic waves

**DOI:** 10.1186/s12916-019-1406-6

**Published:** 2019-08-22

**Authors:** Gerardo Chowell, Amna Tariq, James M. Hyman

**Affiliations:** 1 Department of Population Heath Sciences, School of Public Health, Georgia State University, Atlanta, GA USA; 2 Division of International Epidemiology and Population Studies, Fogarty International Center, National Institutes of Health, Bethesda, MD USA; 3 Department of Mathematics, Center for Computational Science, Tulane University, New Orleans, LA USA

**Keywords:** Mathematical framework, Epidemic wave, Sub-epidemic, Unobserved heterogeneity, SARS, Plague, Ebola, Democratic Republic of Congo, Forecast, Uncertainty, Mean interval score, Reporting delay

## Abstract

**Background:**

Simple phenomenological growth models can be useful for estimating transmission parameters and forecasting epidemic trajectories. However, most existing phenomenological growth models only support single-peak outbreak dynamics whereas real epidemics often display more complex transmission trajectories.

**Methods:**

We develop and apply a novel sub-epidemic modeling framework that supports a diversity of epidemic trajectories including stable incidence patterns with sustained or damped oscillations to better understand and forecast epidemic outbreaks. We describe how to forecast an epidemic based on the premise that the observed coarse-scale incidence can be decomposed into overlapping *sub*-*epidemics* at finer scales. We evaluate our modeling framework using three outbreak datasets: Severe Acute Respiratory Syndrome (SARS) in Singapore, plague in Madagascar, and the ongoing Ebola outbreak in the Democratic Republic of Congo (DRC) and four performance metrics.

**Results:**

The sub-epidemic wave model outperforms simpler growth models in short-term forecasts based on performance metrics that account for the uncertainty of the predictions namely the mean interval score (MIS) and the coverage of the 95% prediction interval. For example, we demonstrate how the sub-epidemic wave model successfully captures the 2-peak pattern of the SARS outbreak in Singapore. Moreover, in short-term sequential forecasts, the sub-epidemic model was able to forecast the second surge in case incidence for this outbreak, which was not possible using the simple growth models. Furthermore, our findings support the view that the national incidence curve of the Ebola epidemic in DRC follows a stable incidence pattern with periodic behavior that can be decomposed into overlapping sub-epidemics.

**Conclusions:**

Our findings highlight how overlapping sub-epidemics can capture complex epidemic dynamics, including oscillatory behavior in the trajectory of the epidemic wave. This observation has significant implications for interpreting apparent noise in incidence data where the oscillations could be dismissed as a result of overdispersion, rather than an intrinsic part of the epidemic dynamics. Unless the oscillations are appropriately modeled, they could also give a false positive, or negative, impression of the impact from public health interventions. These preliminary results using sub-epidemic models can help guide future efforts to better understand the heterogenous spatial and social factors shaping sub-epidemic patterns for other infectious diseases.

**Electronic supplementary material:**

The online version of this article (10.1186/s12916-019-1406-6) contains supplementary material, which is available to authorized users.

## Introduction

The myriad of interrelated, and often unobserved, factors that influence the propagation of pathogens at different spatial and temporal scales create major challenges for predicting the transmission dynamics of infectious disease [1]. The factors influencing infectious disease transmission include the mode of transmission (e.g., close contact, airborne, via vector, sexual route), the individual-level network that captures the dynamics of disease-relevant interactions (which are often influenced by cultural factors) [2], the natural history of the disease, variations in the risk behavior of individuals, reactive public health interventions, the behavior changes in response to an epidemic, and the background immunity of the population shaped by genetic factors and prior exposure to the disease or vaccination campaigns [3–6]. Our ability to generate accurate epidemic forecasts is challenged by the sparse data on the individual- and group-level heterogeneity that affect the dynamics of infectious disease transmission [7–9].

The accuracy of epidemic forecasts is also hindered by the lack of detail in the outbreak incidence and contact data. Usually, forecasting models must be based on aggregated reported incidence cases identified at the onset of symptoms or diagnosis. Epidemic incidence data is a valuable epidemiological tool to assess, and forecast, trends and transmission potential in real time [7, 8, 10–14]. However, the aggregated case data rarely contain the information, such as transmission pathways and other population characteristics, needed to create a realistic model for disease transmission [8]. For example, during the first few months of the 2014–2016 Ebola epidemic in West Africa, weekly national-level epidemic curves for Guinea, Liberia, and Sierra Leone were made publicly available by the World Health Organization (WHO) [11]. In contrast, Ebola virus first affected the village of Gueckedou in Guinea, and the transmission chains rapidly crossed the nearby porous borders of Sierra Leone and Liberia [11]. Therefore, epidemic curves at finer spatial and temporal resolutions covering the relevant interacting communities would have been more pertinent to assess the spreading pattern and guide control efforts.

Limited epidemic data limits the complexity of the mathematical models in terms of the number of mechanisms and parameters that can be estimated from data. These models often use a metapopulation framework to incorporate population heterogeneity by dividing the population into socio-demographic groups based on the susceptibility, infectivity, mobility patterns, or other individual characteristics related to the transmission dynamics [15–18]. The individuals in the same group are assumed to be homogenous, and the heterogeneity of the population is limited by the number of groups. Even when the number of parameters that can be estimated from limited data is small, the model must include enough complexity to account for the underlying transmission dynamics. Past studies indicate that simple logistic-type growth models tend to underestimate the peak timing and duration of epidemic outbreaks [19–21]. Also, these simple logistic-type phenomenological growth models typically can support only a single-wave epidemic trajectory characterized by a single peak in the number of new infections followed by a “burnout” period, unless there are external driving forces, such as a seasonal variation in contact patterns.

We put forward a sub-epidemic modeling framework that supports diverse epidemic wave trajectories, including stable incidence patterns with sustained or damped oscillations. We divide the population into groups, and use overlapping *sub-epidemics* in these groups as the mathematical building blocks to understand and forecast an epidemic observed at coarser scales. Hence, the coarse-scale-observed epidemic is created from the aggregation of overlapping sub-epidemics in the groups that follow a regular structure. These sub-epidemics are usually unobserved and shaped by population heterogeneity. The groups are determined by the susceptibility of the underlying populations (e.g., spatially clustered pockets of susceptible individuals), population mobility patterns, the natural history of the disease, infections moving across different risk groups, varying public health interventions, and rapidly changing environmental factors, to name a few. This approach allows the model forecast to depend upon changes in the composition of individual groups based on temporal changes of healthcare, or local behavior changes that impact the case incidence for a given spatial area or subpopulations such as schools or socio-demographic groups.

In heterogenous populations, the coarse-scale epidemic incidence can rarely be characterized by a simple mathematical function. The overlapping sub-epidemic building block approach helps us understand how to decompose the larger-scale epidemic wave patterns into multiple incidence curves that could be shaped by multiple factors. The coarse-scale epidemic wave can be investigated as the aggregation of regular and overlapping sub-epidemics that are related to each other in some systematic fashion. This reduces the number of free parameters that are necessary to relate sub-epidemics to each other.

After describing the sub-epidemic modeling framework, we will apply the approach to describe and generate short-term forecasts for past outbreaks. In this process, we also systematically compare the goodness of fit and the forecasting performance of the sub-epidemic wave model with that of simpler growth models.

### Mathematical framework of epidemic waves composed of overlapping sub-epidemics

We model each group sub-epidemic by a generalized-logistic growth model (GLM) which has displayed promising performance for short-term forecasting the trajectory of emerging infectious disease outbreaks [20–22]. The GLM is given by the following differential equation:

$$ \frac{dC(t)}{dt}={rC}^p(t)\left(1-\frac{C(t)}{K_0}\right) $$

where $\frac{dC(t)}{dt}$ describes the incidence curve over time *t*. The cumulative number of cases at time *t* is given by *C*(*t*), while *r* is a positive parameter denoting the growth rate ((people)^1 − *p*^ per time), *K*_0_ is the final epidemic size, and *p* ∈ [0, 1] is the “scaling of growth” parameter. If *p* = 0, this equation describes a constant incidence over time, while if *p* = 1 the equation becomes the well-known exponential growth model. Intermediate values of *p*(0 < *p* < 1) describe sub-exponential (e.g., polynomial) growth patterns.

**Fig. 1:**
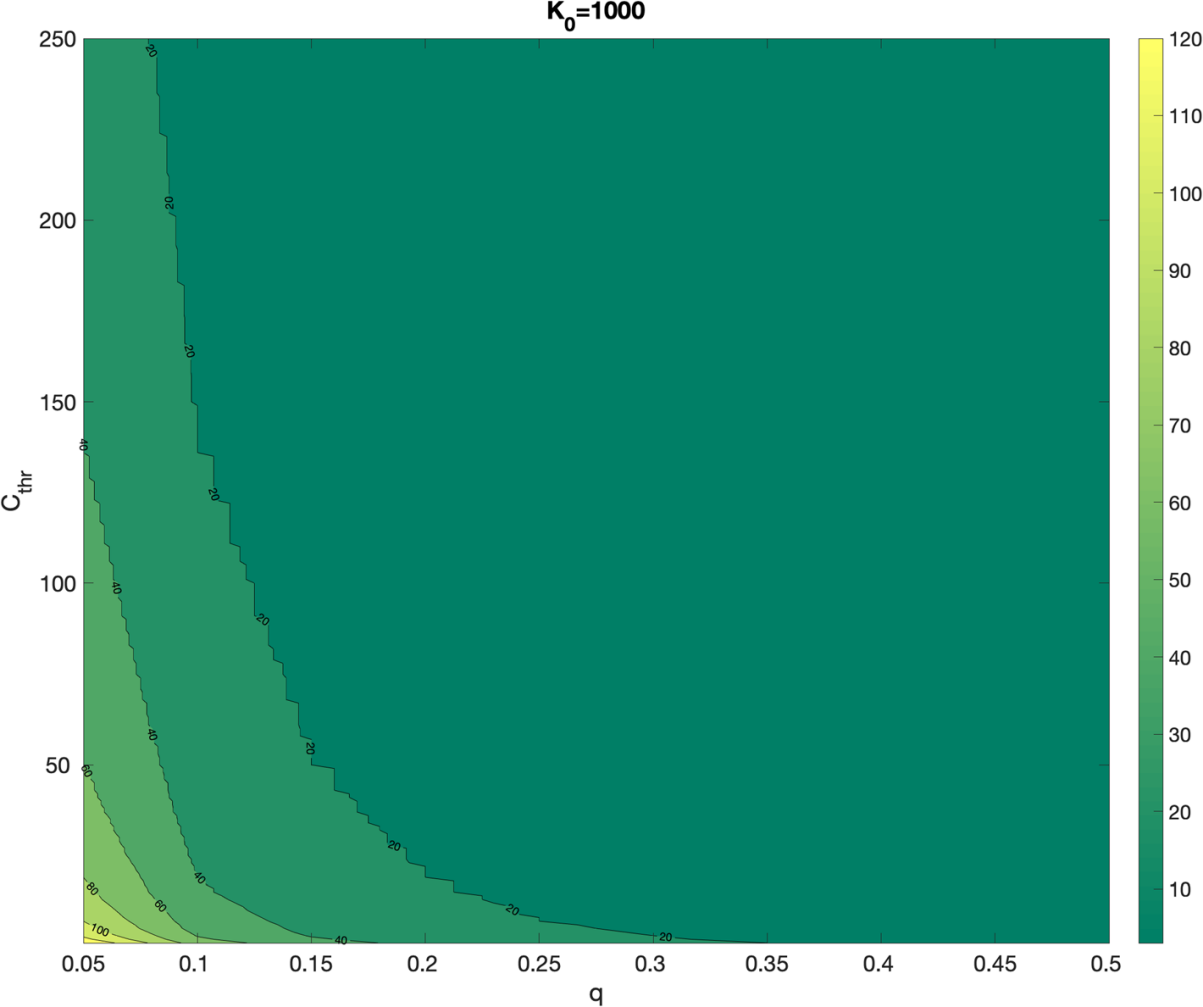
The number of sub-epidemics for epidemic waves associated with different parameters. The number of sub-epidemics comprising an epidemic wave depends on the parameters *K*_0_, *q*, and *C*_thr_ as explained in the main text

Next, we model an epidemic wave comprising a set of *n* overlapping sub-epidemics that follow a regular structure using the following system of coupled differential equations:

$$ \frac{dC_i(t)}{dt}={rA}_{i-1}(t){C}_i{(t)}^p\left(1-\frac{C_i(t)}{K_i}\right) $$

where *C*_*i*_(*t*) tracks the cumulative number of infections for sub-epidemic *i* and *K*_*i*_ is the size of the *i*th sub-epidemic where *i* = 1…*n*. Thus, the model reduces to the simple logistic-type model when *n* = 1. To model the onset timing of the (*i* + 1)_th_ sub-epidemic, we employ an indicator variable given by *A*_*i*_(*t*) so that the sub-epidemics comprising an epidemic wave follow a *regular* structure because the (*i* + 1)_th_ sub-epidemic is triggered when the cumulative number of cases for the *i*th sub-epidemic exceeds a total of *C*_thr_ cases and *overlapping* because the (*i* + 1)_th_ sub-epidemic takes off before the *i*th sub-epidemic completes its course. Hence,

$$ {A}_i(t)=\left\{\begin{array}{cc}1& {C}_i(t)>{C}_{\mathrm{thr}}\\ {}0& \mathrm{Otherwise}\end{array}i=1,2,3,\dots n\kern0.5em \right. $$

where 1 ≤ *C*_thr_ < *K*_0_ and *A*_1_(*t*) = 1 for the first sub-epidemic. Moreover, the size of the *i*th sub-epidemic (*K*_*i*_) declines exponentially at rate *q* for subsequently occurring sub-epidemics due to multiple factors including seasonal transmission effects, a gradually increasing effect of public health interventions or population behavior changes that mitigate transmission. If *q* = 0, the model predicts an epidemic wave comprising sub-epidemics of the same size. Note that alternative decline functions could be considered such as harmonic or hyperbolic decline functions. Assuming that subsequent sub-epidemic sizes decline exponentially, we have:

$$ {K}_i={K}_0{e}^{-q\left(i-1\right)} $$

where *K*_0_ is the size of the initial sub-epidemic (*K*_1_ = *K*_0_). Hence, when *q* > 0, the total number of sub-epidemics supported by the model depends on *C*_thr_, *q*, and, *K*_0_ because the (*i* + 1)_th_ sub-epidemic is only triggered if *C*_thr_ ≤ *K*_*i*_ (Fig. 1). Moreover, the total size of an epidemic wave composed of *n* overlapping sub-epidemics is simply given by:

$$ {K}_{\mathrm{tot}}=\sum \limits_{i=1}^n{K}_0{e}^{-q\left(i-1\right)}=\frac{K_0\left(1-{e}^{- qn}\right)}{1-{e}^{-q}} $$

In the absence of control interventions or behavior change (*q* = 0), the total epidemic size is given by:

$$ {K}_{\mathrm{tot}}={nK}_0 $$

The initial number of cases given by *C*_1_(0) = *I*_0_ where *I*_0_ is the initial number of cases in observed case data. Then, the cumulative curve of the epidemic wave denoted by *C*_tot_(*t*) is obtained by aggregating all of the *n* overlapping sub-epidemics comprising the epidemic wave:

$$ {C}_{\mathrm{tot}}(t)=\sum \limits_{i=1}^n{C}_i(t) $$

### Epidemic wave profiles

We use our model to characterize five broad profiles of overlapping sub-epidemics shaping epidemic waves: (1) stationary endemic waves, (2) single-peak epidemic waves composed of a finite number of sub-epidemics with or without the mitigative effects of control interventions and/or behavior changes, and (3) epidemic waves with oscillatory behavior composed of a finite number of sub-epidemics with or without the mitigative effects of control interventions and/or behavior changes.

### Parameter uncertainty and identifiability

Lack of identifiability or non-identifiability arises when one or more parameter estimates are associated with large uncertainties. This may be attributed to the model structure (structural identifiability) or due to the lack of information in a given dataset, which could be associated with the number of observations and the spatial-temporal resolution of the data [23, 24]. Because the time series of incident cases in the observed epidemic wave is an aggregation of the overlapping sub-epidemics, different sub-epidemic profiles may give rise to indistinguishable aggregated epidemic waves. This can happen if the parameters are correlated and different combinations of parameters result in the same fit of the data but have different forecasts. For a given epidemic wave, the number of sub-epidemics could be correlated with the size of individual sub-epidemics and parameter *C*_thr_ which sets the timing of the subsequent sub-epidemic. For example, given a fixed sub-epidemic size, as *C*_thr_ increases, a smaller number of sub-epidemics can be fit to the epidemic wave.

When a parameter is associated with substantial uncertainty, researchers may decide to constrain its range to lie within a plausible or realistic range and as close as possible to a best guess based on demographic and epidemiological data. For instance, the size of the first sub-epidemic should not be too small (e.g., *K*_0_ > 100). Moreover, by design the number of sub-epidemics comprising an epidemic wave in our model is constrained by *C*_thr_ < *K*_0_. Further, the cumulative case threshold *C*_thr_ could be further constrained so that it does not exceed the cumulative number of cases at peak incidence.

### Parameter estimation

Our parameter estimation approach has been described in prior publications (e.g., [19, 25]). Calibrating our sub-epidemic modeling framework to time series data requires estimating 5 model parameters namely Θ = (*C*_thr_, *q*, *r*, *p*, *K*). Model parameters were estimated via least-square fitting of the model solution to the observed incidence data [26]. This is achieved by searching for the set of parameters $\hat{\Theta}=\left({\hat{\theta}}_1,{\hat{\theta}}_2,\dots, {\hat{\theta}}_m\right)$ that minimize the sum of squared differences between the observed incidence data ${y}_{t_i}={y}_{t_1},{y}_{t_1},\dots, {y}_{t_n}$ and the corresponding mean incidence curve denoted by *f*(*t*_*i*_, Θ). That is, the objective function is given by

$$ \hat{\Theta}=\arg \min \sum \limits_{i=1}^n{\left(f\left({t}_i,\Theta \right)-{y}_{t_i}\right)}^2 $$

where *t*_*i*_ are the time points at which the time series data are observed, and *n* is the number of data points available for inference. Hence, the model solution $f\left({t}_i,\hat{\Theta}\right)$ yields the best fit to the time series data ${y}_{t_i}$. We solve the nonlinear least squares problem using the trust-region reflective algorithm. We used parametric bootstrap, assuming a Poisson error structure, to quantify the uncertainty in the parameters obtained by a nonlinear least squares fit of the data, as described in refs. [19, 25]. Our best-fit model solution is given by $f\left(t,\hat{\Theta}\right)$ where $\hat{\Theta}$ is the vector of parameter estimates. Our MATLAB (The Mathworks, Inc) code for model fitting along with outbreak datasets is publicly available [27].

The model confidence intervals of the parameters and 95% prediction intervals of the model fits were obtained using parametric bootstrap [19]. That is, we re-estimated the parameters ${\hat{\Theta}}_i$ where *i* = 1, 2, …, *S*. Here, *S* is the number of bootstrap realizations, and the uncertainty around the mean of model fit is defined by $f\left(t,{\hat{\Theta}}_1\right),f\left(t,{\hat{\Theta}}_2\right),\dots, f\left(t,{\hat{\Theta}}_S\right)$. This information can be further used to generate 95% prediction intervals. Note that these model confidence intervals are for the model, not the true underlying epidemic. Since the model is only an approximation of the underlying transmission dynamics, the model discrepancy can result in the observations and forecasts that are outside these model confidence intervals. The uncertainty of the model forecasts, $f\left(t,\hat{\Theta}\right)$, is estimated using the variance of the parametric bootstrap samples

$$ f\left(t,{\hat{\Theta}}_1\right),f\left(t,{\hat{\Theta}}_2\right),\dots, f\left(t,{\hat{\Theta}}_S\right) $$

where ${\hat{\Theta}}_i$ denotes the estimation of parameter set Θ from the *i*th bootstrap sample. The 95% prediction intervals of the forecasts in the examples are calculated from the 2.5% and 97.5% percentiles of the bootstrap forecasts.

### Assessing model performance

In order to evaluate the performance of our sub-epidemic wave model in its capacity to describe and forecast incidence patterns in the short term, we compared it to the well-known two-parameter logistic growth model and the three-parameter Richards model [28, 29]. While the logistic growth model is nested within our sub-epidemic modeling framework, the Richards model is not. The logistic growth model (LM) is given by:

$$ \frac{dC(t)}{dt}= rC(t)\left(1-\frac{C(t)}{K_0}\right) $$

The Richards model with three parameters (*r*, *a*, *K*) is given by:

$$ \frac{dC(t)}{dt}= rC(t)\left(1-{\left(\frac{C(t)}{K_0}\right)}^a\right) $$

where the parameter *a* is a positive constant.

To assess both the quality of the model fit and the short-term forecasts, we employed four performance metrics: the mean absolute error (MAE), the mean squared error (MSE), the coverage of the 95% prediction intervals, and the mean interval score (MIS) [30].

The mean absolute error (MAE) is given by:

$$ \mathrm{MAE}=\frac{1}{n}\sum \limits_{i=1}^n\left|f\left({t}_i,\hat{\Theta}\right)-{y}_{t_i}\right| $$

Here ${y}_{t_i}$ is the time series of incident cases describing the epidemic wave where *t*_*i*_ are the time points of the time series data [31]. Similarly, the mean squared error (MSE) is given by:

$$ \mathrm{MSE}=\frac{1}{n}\sum \limits_{i=1}^n{\left(f\left({t}_i,\hat{\Theta}\right)-{y}_{t_i}\right)}^2 $$

In addition, we assessed the *coverage of the 95% prediction interval*, e.g., the proportion of the observations that fell within the 95% prediction interval and a metric that addresses the width of the 95% prediction interval as well as coverage via the *mean interval score* (MIS) [30, 32] which is given by:

$$ \mathrm{MIS}=\frac{1}{h}\sum \limits_{i=1}^h\left({U}_{t_i}-{L}_{t_i}\right)+\frac{2}{0.05}\left({L}_{t_i}-{y}_{t_i}\right)\mathrm{I}\left\{{y}_{t_i}<{L}_{t_i}\right\}+\frac{2}{0.05}\left({y}_{t_i}-{U}_{t_i}\right)\mathrm{I}\left\{{y}_{t_i}>{U}_{t_i}\right\} $$

where *L*_*t*_ and *U*_*t*_ are the lower and upper bounds of the 95% prediction interval and Ι{} is an indicator function. Thus, this metric rewards for narrow 95% prediction intervals and penalizes at the points where the observations are outside the bounds specified by the 95% prediction interval where the width of the prediction interval adds up to the penalty (if any) [30].

The mean interval score and the coverage of the 95% prediction intervals take into account the uncertainty of the predictions whereas the MAE and MSE only assess the closeness of the mean trajectory of the epidemic to the observations [8]. These performance metrics have also been adopted in international forecasting competitions [32].

### Application to epidemic outbreaks

We apply our modeling framework to describe and short-term forecast three real outbreaks namely severe acute respiratory syndrome (SARS) in Singapore, plague in Madagascar, and the ongoing Ebola outbreak in the Democratic Republic of Congo (DRC).

#### SARS outbreak in Singapore

We obtained the daily number of new SARS cases by date of symptom onset of the 2003 SARS outbreak in Singapore [33] (Additional file 1). This outbreak involved three major hospitals in Singapore, and the incidence curve exhibited two overlapping waves that peaked in mid-March and early April (2003), respectively. These two small waves largely correspond to sub-epidemics stemming from different healthcare settings [33]. This epidemic lasted a total of 70 days. For each model, we generated a total of 46 short-term forecasts from day 15 until day 60. We evaluated 4, 6, 8, and 10 days ahead forecasts.

#### Ebola outbreak in DRC, September, 2018, to mid-April, 2019

We obtained a weekly incidence curve according to the date of symptom onset for the second wave of the ongoing Ebola outbreak in the DRC from the WHO Situation Reports and Disease Outbreak News covering the reporting period: September 2018 to mid-April 2019 [34]. The incidence curve of the epidemic was further adjusted for reporting delays as described in ref. [35]. Briefly, the curve of crude incidence by date of symptom onset was adjusted for reporting delays using a nonparametric method that adapts survival analysis and life table techniques for use with right truncated data [36, 37]. This epidemic has become the second largest Ebola outbreak in history with 1186 reported cases as of April 11, 2019, despite active ring vaccination efforts in the region [34]. The outbreak was first reported on August 1, 2018, by the WHO, spreading in the urban areas of the provinces of North Khivu and Ituri in the northeast region that borders Uganda [38]. Unfortunately, armed conflict in the Ebola-affected zone is hindering rapid response activities including case detection, contact tracing, isolation, and vaccination. Prolonged transmission has been primarily attributed to poor infection control practices in healthcare settings, delays in case detection and isolation, community resistance, and violent attacks targeting health workers and health centers [38]. For each model, we conducted a total of 19 forecasts from week 8 to week 26 of the epidemic. We assessed 2, 3, 4, and 5 weeks ahead forecasts.

#### Plague outbreak in Madagascar

We analyzed the main epidemic wave of the 2017 plague epidemic in Madagascar which was retrieved from the WHO reports. The epidemic wave consists of weekly confirmed, probable and suspected plague cases during September–November 2017 [39]. The epidemic comprises 50 incidence weeks. For each model, we generated a total of 26 forecasts from week 10 to week 35 of the epidemic. We assessed 2, 3, 4, and 5 weeks ahead forecasts.

## Results

**Fig. 2:**
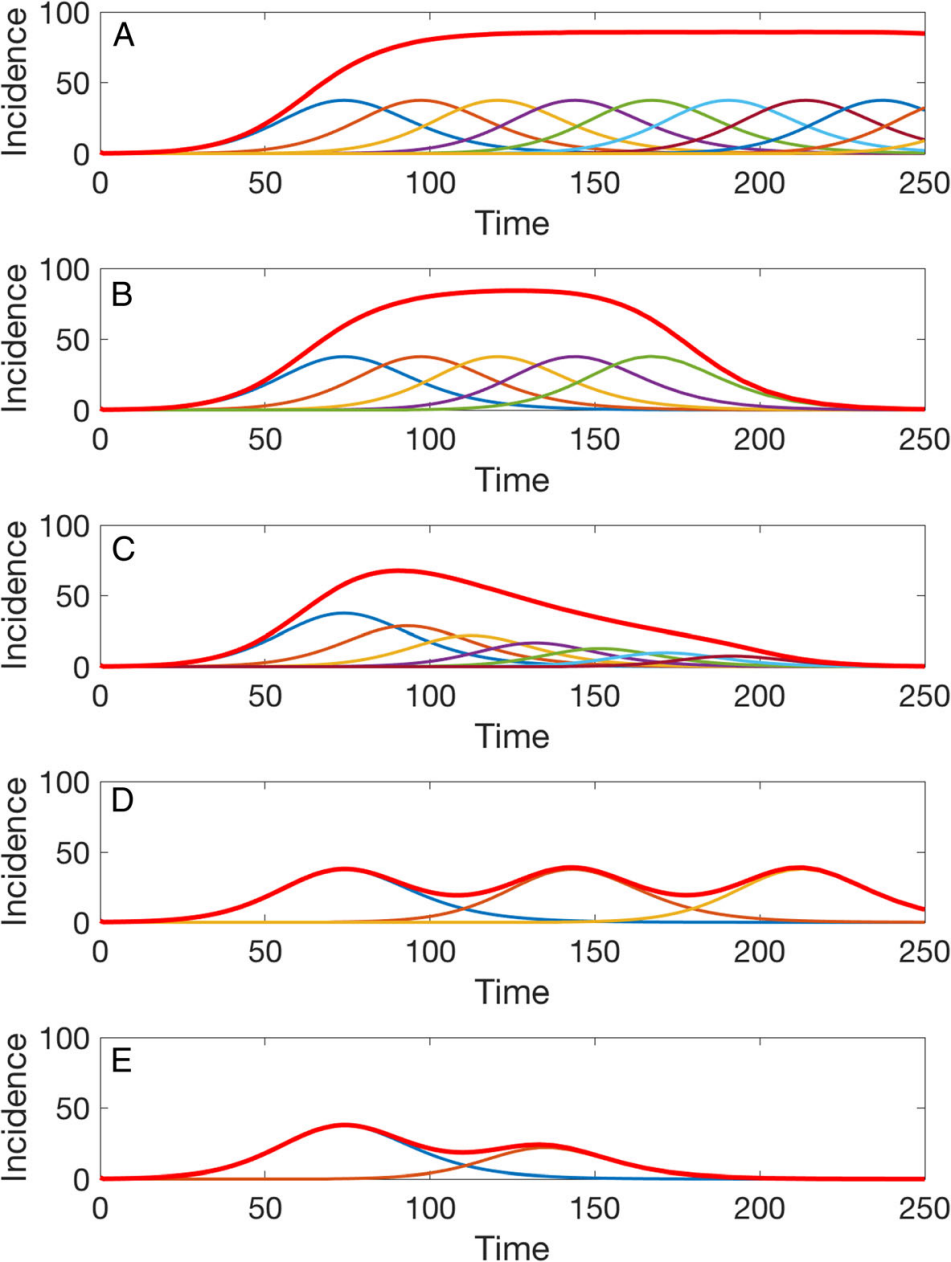
Epidemic wave profiles or the taxonomy of overlapping sub-epidemic waves. Five representative epidemic waves comprised of sub-epidemics characterized by the following parameters: *r* = 0.15, *p* = 0.9, *K* = 2000. **a** The first panel shows a stationary 300-day *endemic wave* comprising 10 sub-epidemics with a cumulative case threshold *C*_thr_ of 20 cases and parameter *q* = 0. **b** The second panel displays a *temporary endemic* wave comprising 5 sub-epidemics with a cumulative case threshold *C*_thr_ of 20 cases and parameter *q* = 0. **c** The third panel shows an epidemic wave comprising 10 declining sub-epidemics with a cumulative case threshold *C*_thr_ of 20 cases where subsequent sub-epidemics decline exponentially with rate *q* = 0.3. **d** The fourth panel displays an epidemic wave with *sustained oscillations* composed of three sub-epidemics with a high cumulative case threshold *C*_thr_ of 800 without the effects of interventions (*q* = 0). **e** Finally, the fifth panel shows an epidemic wave with *damped oscillations* comprising two sub-epidemics where the second one is affected by interventions or behavior changes (*q* = 0.6) and is triggered once the first one has accumulated 40% of its total size

Figure 2 displays five representative epidemic waves comprised of overlapping sub-epidemics characterized by the following parameters: *r* = 0.15, *p* = 0.9, *K* = 2000. Specifically, the first panel shows a stationary 300-day endemic wave comprising 10 sub-epidemics with a cumulative case threshold *C*_thr_ of 20 cases and parameter *q* = 0. Note that the last few sub-epidemics have not completed their course by the end of the simulation period. The second panel displays a temporary endemic wave comprising 5 sub-epidemics with a cumulative case threshold *C*_thr_ of 20 cases and parameter *q* = 0. This epidemic wave profile differs from the previous one in that all of the sub-epidemics have completed their course within the first 250 days of the simulation. The third panel shows an epidemic wave comprising 10 declining sub-epidemics with a cumulative case threshold *C*_thr_ of 20 cases where subsequent sub-epidemics declines exponentially with rate *q* = 0.3. The fourth panel displays an epidemic wave with sustained oscillations composed of three sub-epidemics with a high cumulative case threshold *C*_thr_ of 800 without the effects of interventions (*q* = 0). That is, each new sub-epidemic is not triggered until the previous sub-epidemic has accumulated 90% of its final sub-epidemic size. Finally, the fifth panel represents an epidemic wave with damped oscillations comprising two sub-epidemics where the second one is affected by interventions or behavior changes (*q* = 0.6) and is triggered once the first one has accumulated 40% of its total size.

### Quality of the model fits to outbreak data

**Fig. 3:**
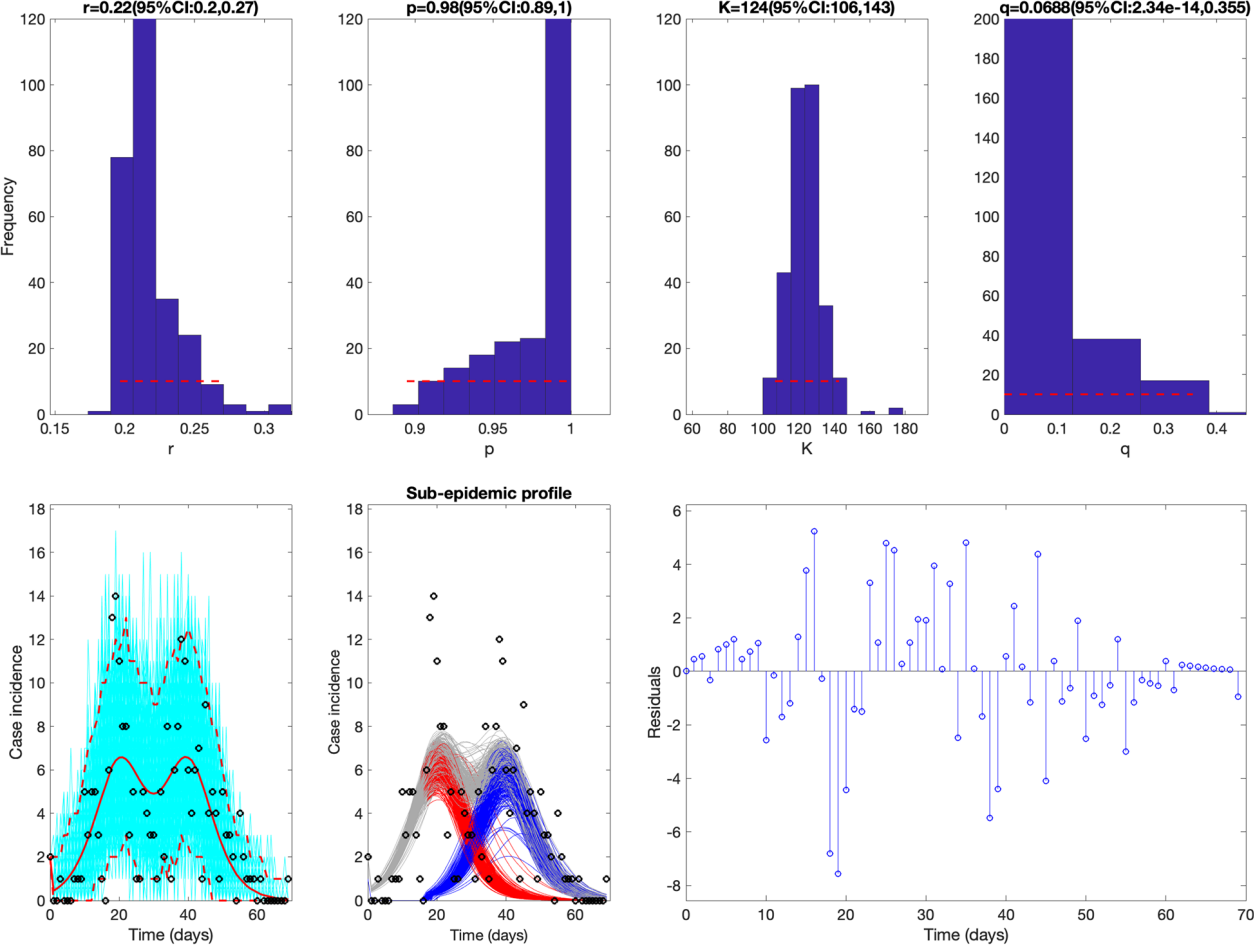
Best fit of the sub-epidemic model to the SARS outbreak in Singapore. Our sub-epidemic model yielded the best fit to the incidence curve of the SARS outbreak (Table 1). Moreover, the model successfully predicts the 2-wave pattern of the outbreak. Further, parameter estimates are well identified as indicated by their relatively narrow confidence intervals. For instance, the 95% confidence interval for the size of the initial sub-epidemic ranges between 106 and 143 cases. The top panels display the empirical distribution of *r*, *p*, *K*, and *q*. Bottom panels show the model fit (left), the sub-epidemic profile (center), and the residuals (right). Black circles correspond to the data points. The best model fit (solid red line) and 95% prediction interval (dashed red lines) are also shown. Cyan curves are the associated uncertainty from individual bootstrapped curves assuming a Poisson error structure. Different sub-epidemics comprising the epidemic wave are plotted using different colors

**Fig. 4:**
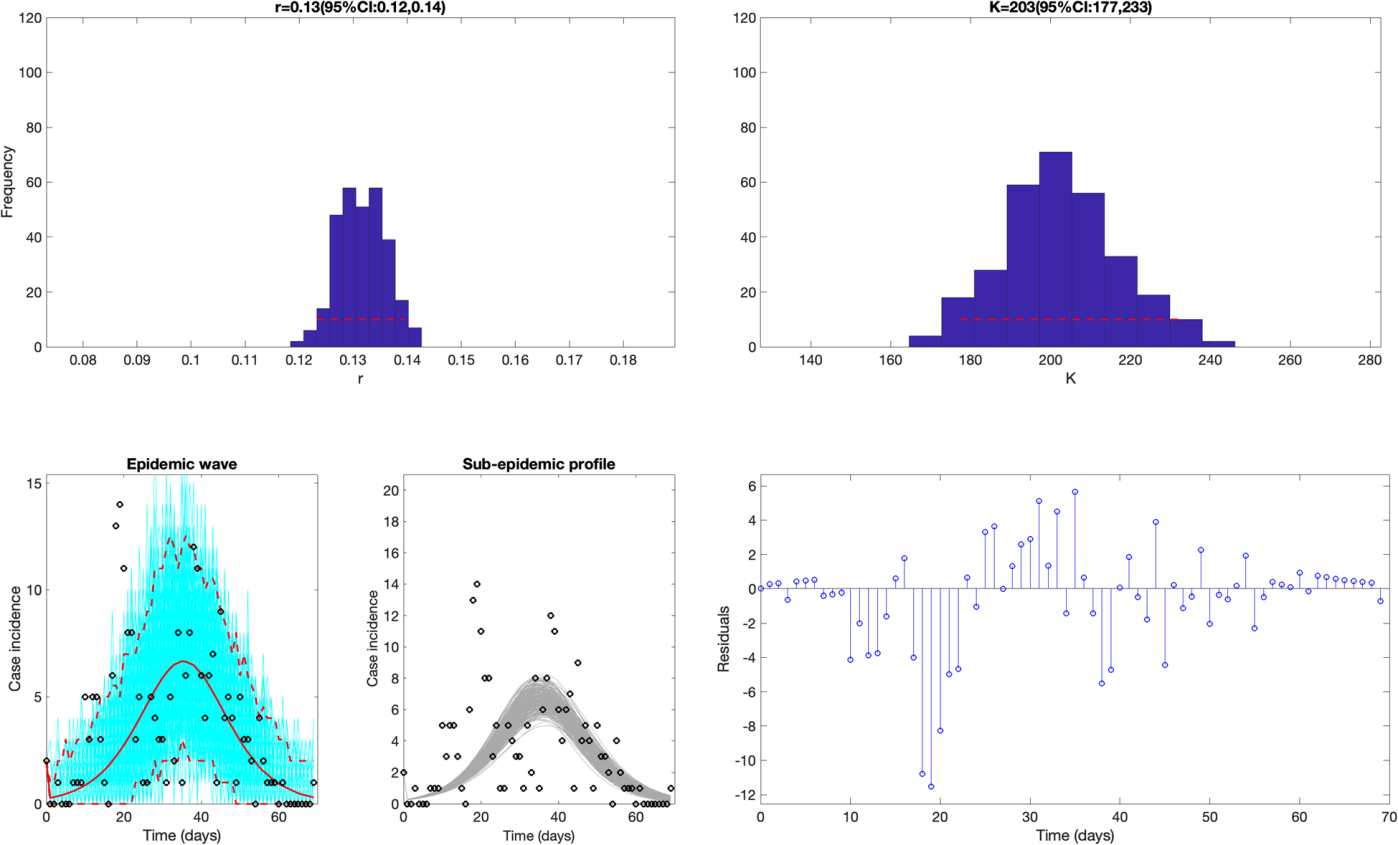
Fit of the simple logistic growth model to the SARS outbreak in Singapore. This simple model was unable to reproduce the bimodal shape of the outbreak. The top panels display the empirical distribution of *r* and *K*. Bottom panels show the model fit (left), the sub-epidemic profile (center), and the residuals (right). Black circles correspond to the data points. The best model fit (solid red line) and 95% prediction interval (dashed red lines) are also shown. Cyan curves are the associated uncertainty from individual bootstrapped curves assuming a Poisson error structure

**Fig. 5:**
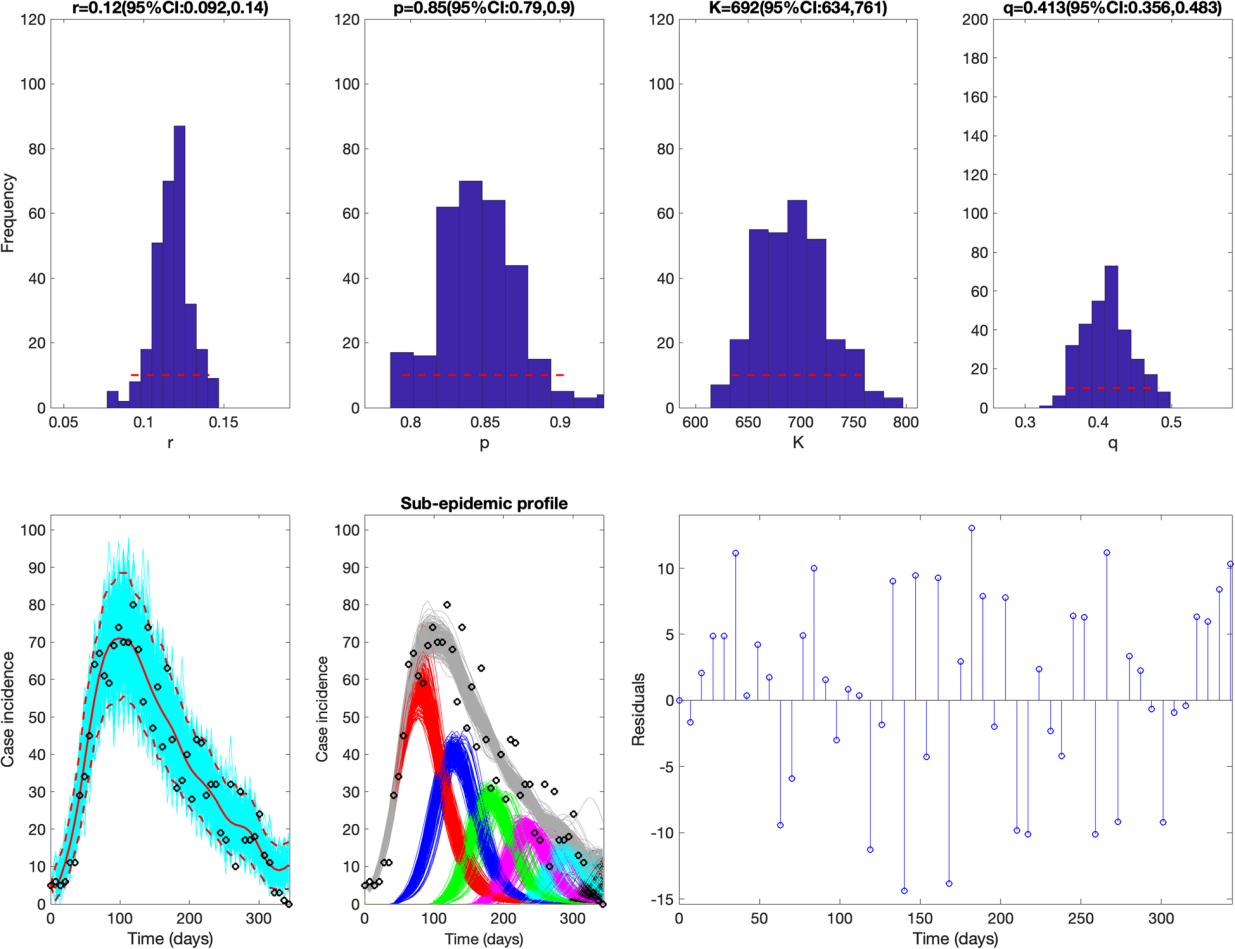
Best fit of the sub-epidemic wave model to the plague epidemic in Madagascar. This model yielded the best fit to the weekly incidence curve. Moreover, our results predict an epidemic wave comprised by 5 sub-epidemics of decreasing size. Further, parameter estimates are well identified as indicated by their relatively narrow confidence intervals. For instance, the 95% confidence interval for the size of the initial sub-epidemic ranges between 634 and 761. The top panels display the empirical distribution of the parameter estimates (*r*, *p*, *K*, and *q*). Bottom panels show the model fit (left), the sub-epidemic profile (center), and the residuals (right). Black circles correspond to the data points. The best model fit (solid red line) and 95% prediction interval (dashed red lines) are also shown. Cyan curves are the associated uncertainty from individual bootstrapped curves assuming a Poisson error structure. Different sub-epidemics comprising the epidemic wave are plotted using different colors

The sub-epidemic model consistently yielded the best fit to the daily incidence curves for each of the three outbreaks (SARS, plague, and Ebola) based on the four performance metrics (MAE, MSE, MIS, and the coverage of the 95% prediction interval) as shown in Table 1. For the SARS outbreak in Singapore, the sub-epidemic model was able to successfully capture the two-wave pattern of the SARS outbreak, and the model parameter estimates were well identified (Fig. 3). In contrast, the simpler single-peak growth models were unable to reproduce the bimodal shape of the outbreak, yielding poorer performance (Table 1 and Fig. 4). For the plague outbreak in Madagascar, the sub-epidemic model also outperformed the other simple models (Table 1) and captured an epidemic wave comprised by 5 sub-epidemics of decreasing size (Fig. 5). Further, parameter estimates for this outbreak were also well identified as indicated by their relatively small uncertainty (Fig. 5). For instance, the 95% confidence interval for the size of the initial sub-epidemic ranges between 634 and 761.

**Table 1:** Quality of the model fits to outbreak data. The sub-epidemic model yielded the best fit to the daily incidence curves based on four performance metrics described in the text. Values highlighted in italics correspond to the best performance metric for a given outbreak

Model	Mean absolute error (MAE)	Mean squared error (MSE)	Mean interval score (MIS)	Percentage coverage of the 95% prediction interval
SARS in Singapore				
Sub-epidemic wave	*1.7*	*6.2*	*10.9*	*90.0*
Richards	2.1	8.1	15.5	85.7
Logistic	2.0	9.8	22.7	84.3
Plague in Madagascar				
Sub-epidemic wave	*5.9*	*50.8*	*37.7*	*80*
Richards	7.0	77.7	63.0	70
Logistic	16.4	408.6	452.0	26
Ebola in DRC				
Sub-epidemic wave	*8.0*	*117.8*	*89.6*	*75.0*
Richards	13.2	276.2	251.6	40.6
Logistic	18.1	467.7	463.2	28.1

**Fig. 6:**
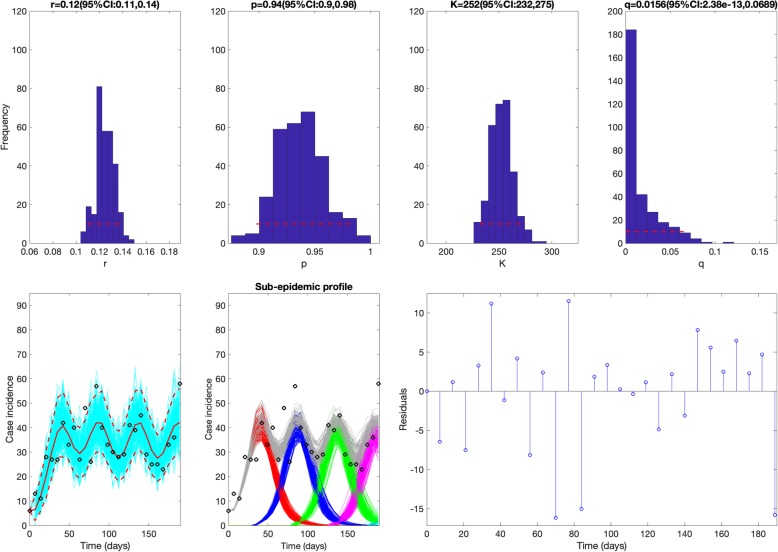
Best fit of the sub-epidemic model to the Ebola epidemic in DRC. Based on the first 28 weeks of the Ebola epidemic in DRC (06 Sep 2018 to 11 Mar 2019), our sub-epidemic model yielded the best fit to the incidence curve. Moreover, our results predict an epidemic wave comprised by 4 sub-epidemics of stable size (~ 250 cases) as the parameter *q* is estimated to be very low, suggesting a stable incidence pattern. Further, parameter estimates are well identified as indicated by their relatively narrow confidence intervals. For instance, the 95% confidence interval for the sub-epidemic size ranges from 232 to 275. The top panels display the empirical distribution of *r*, *p*, *K*, and *q*. Bottom panels show the model fit (left), the sub-epidemic profile (center), and the residuals (right). Black circles correspond to the data points. The model fit (solid red line) and 95% prediction interval (dashed red lines) are also shown. Cyan curves are the associated uncertainty from individual bootstrapped curves assuming a Poisson error structure. Different sub-epidemics of the epidemic wave profile are plotted using different colors

During the first 28 weeks of the ongoing Ebola epidemic in DRC (06 Sep 2018 to 11 Mar 2019), our sub-epidemic model outperformed the simpler models (Table 1 and Fig. 6). For instance, the sub-epidemic model yielded a much lower MIS and much higher coverage of the 95% prediction interval compared to simpler growth models (Table 1). Moreover, our results predict an epidemic wave consisting of 4 sub-epidemics of stable size (~ 250 cases) as the parameter *q* is estimated to be very low, suggesting a stable incidence pattern (Fig. 6). Further, parameter estimates are well identified as indicated by their relatively narrow confidence intervals. For instance, the 95% confidence interval for the initial sub-epidemic size ranges from 232 to 275. However, some of the most recent incidence data points lie substantially above the upper bound of the 95% prediction interval. These anomalies suggest that substantial changes in the underlying dynamics (beyond stochasticity) have occurred more recently.

### Short-term forecasting performance

**Fig. 7:**
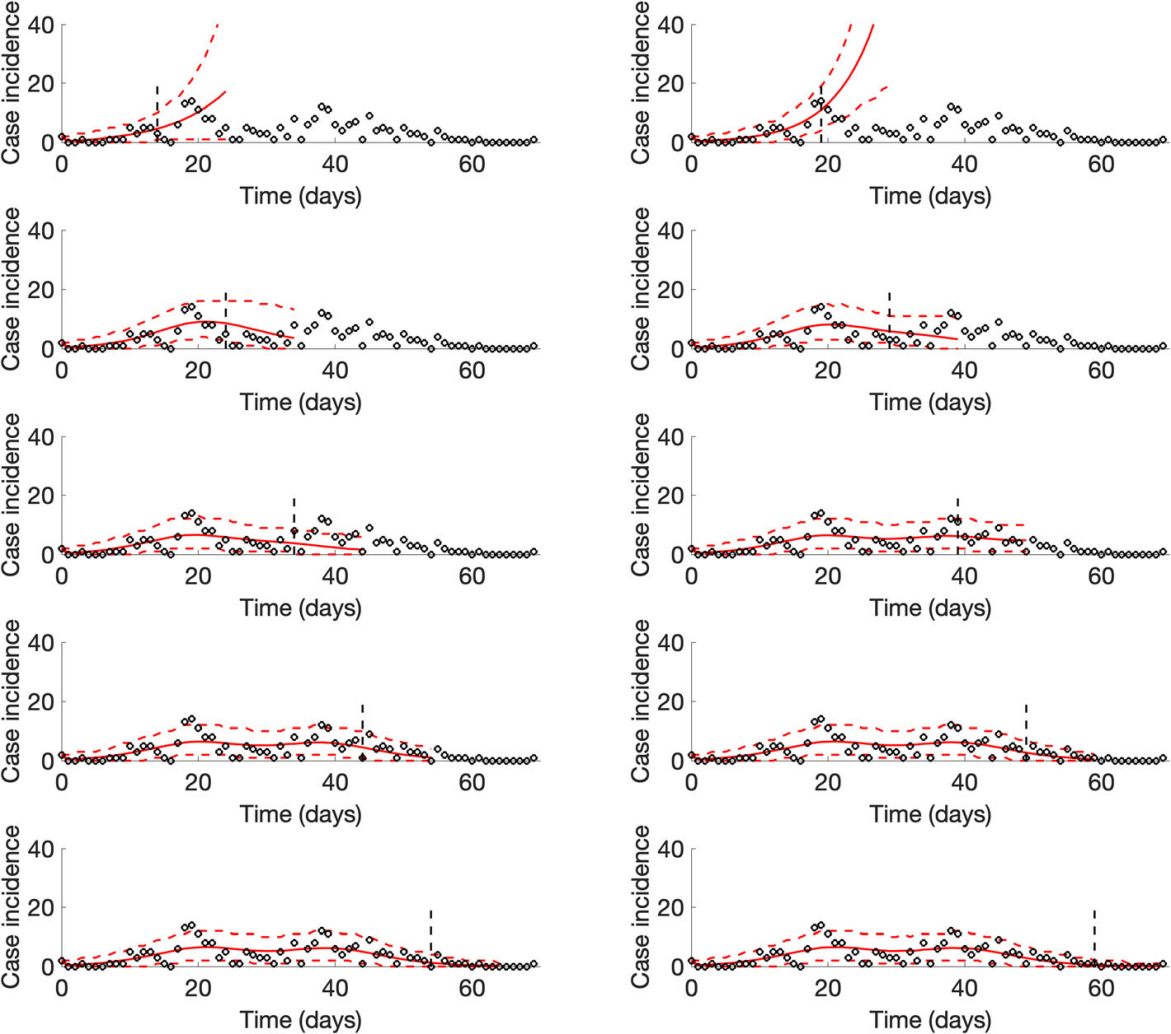
Representative 10-day ahead forecasts of the sub-epidemic model to the SARS outbreak in Singapore. The model was able to capture the two-wave pattern once the model is calibrated using data that includes the early surge of the second sub-epidemic. Black circles correspond to the data points. The model fit (solid red line) and 95% prediction interval (dashed red lines) are also shown. The vertical line indicates the start time of the forecast

For the SARS outbreak in Singapore, our sub-epidemic model outperformed the simpler growth models in terms of the mean interval score and the coverage of the 95% prediction interval across the 4, 6, 8, and 10 days ahead short-term forecasts (Table 2). However, at longer forecast horizons (8 and 10 days), the MAE and the MSE tended to be lower for the Richards model. Unlike the sub-epidemic model (Fig. 7), the simpler models were unable to forecast the second surge in case incidence of the SARS outbreak. Further, the quality of the fit provided by the simpler models during the calibration period deteriorates as the number of data points increases.

**Table 2:** Short-term forecasting performance in the context of the SARS outbreak in Singapore. The sub-epidemic model outperformed the simpler growth models in terms of all of the performance metrics in short-term forecasts. Values highlighted in italics correspond to the best performance metric at a given forecasting horizon

Model	Mean absolute error (MAE)	Mean squared error (MSE)	Mean interval score (MIS)	Percentage coverage of the 95% prediction interval
4 days ahead				
Sub-epidemic wave	*3.6*	*28.1*	*40.6*	*76.1*
Richards	3.7	28.8	79.1	63.3
Logistic	3.8	31.1	60.3	69.4
6 days ahead				
Sub-epidemic wave	*4.0*	*39.5*	*46.9*	*76.3*
Richards	4.1	39.7	87.9	60.4
Logistic	4.1	42.0	66.0	69.3
8 days ahead				
Sub-epidemic wave	4.4	55.7	*54.1*	*75.6*
Richards	*4.4*	*54.5*	94.7	59.4
Logistic	4.4	56.9	71.1	68.9
10 days ahead				
Sub-epidemic wave	4.9	83.5	*60.3*	*74.0*
Richards	*4.8*	*79.3*	99.0	58.9
Logistic	4.8	81.7	77.2	68.0

**Fig. 8:**
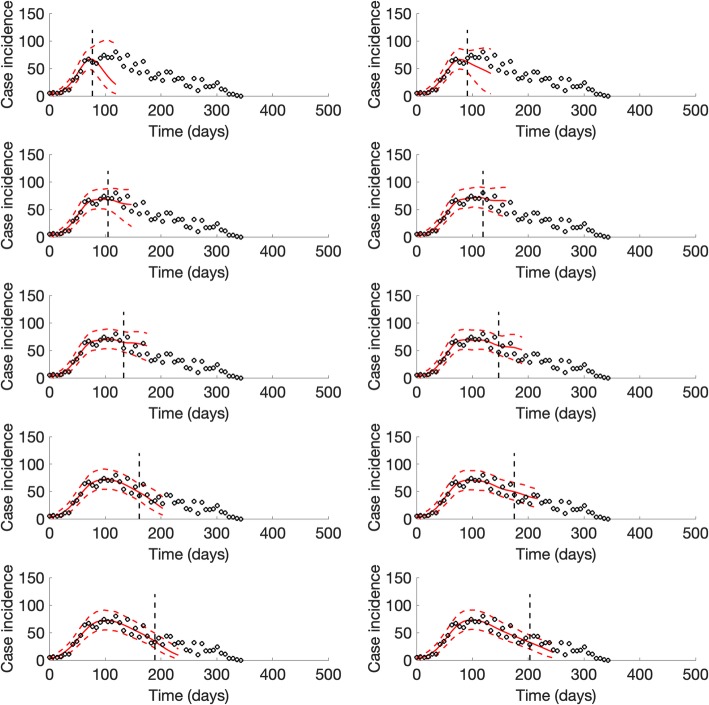
Representative 5-week ahead forecasts of the sub-epidemic model to the plague outbreak in Madagascar. The model was able to outperform simpler growth models in short-term forecasts based on the MAE, the MIS, and the coverage of the 95% prediction interval (Table 3). Black circles correspond to the data points. The model fit (solid red line) and 95% prediction interval (dashed red lines) are also shown. The vertical line indicates the start time of the forecast

**Fig. 9:**
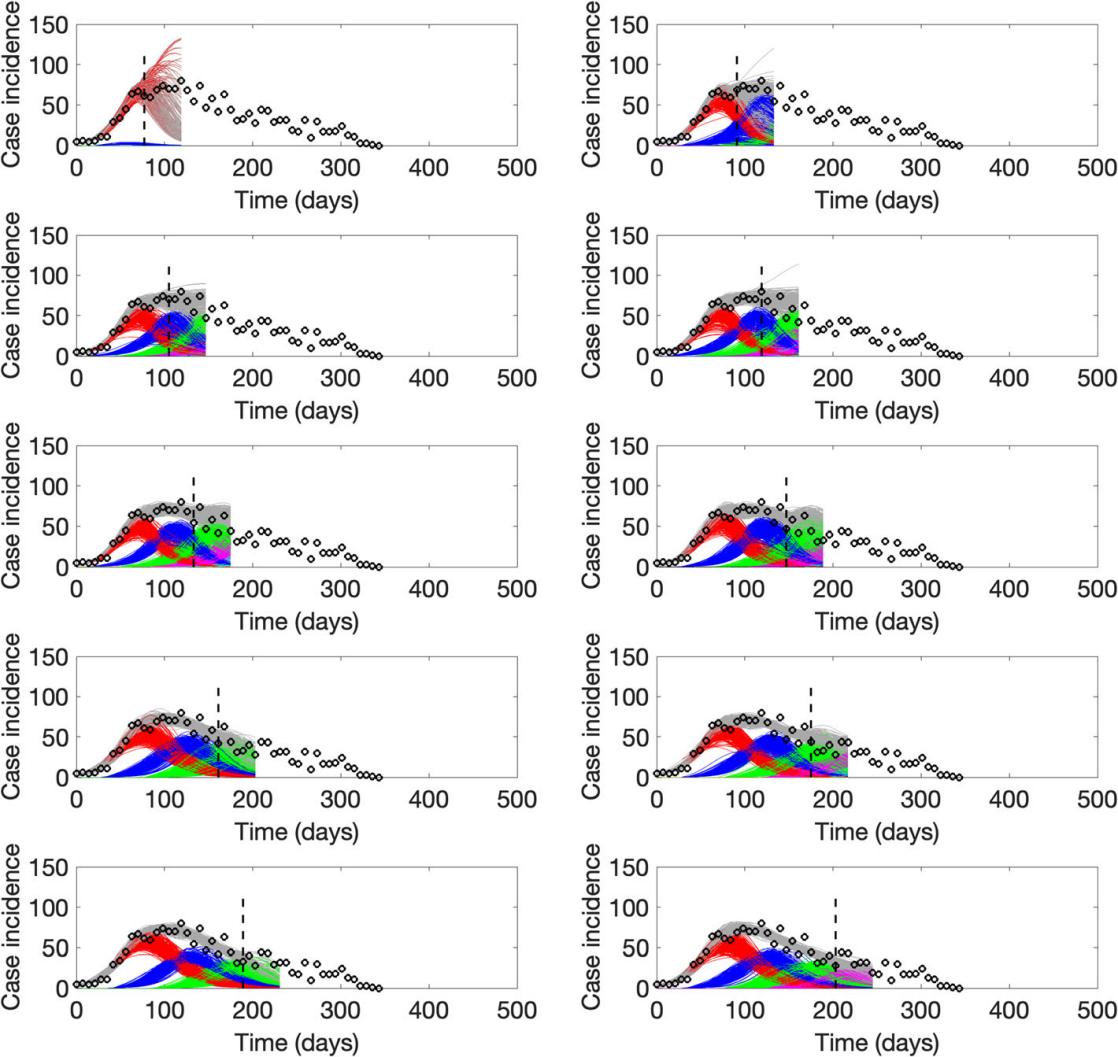
Sub-epidemic profiles of the epidemic wave forecasts for the plague epidemic in Madagascar displayed in Fig. 8. The epidemic wave model predicts a “traveling wave” composed of asynchronous sub-epidemics when the model is fitted to the weekly incidence just before or around the epidemic peak. Once the declining phase of the epidemic is apparent, the model predicts a slowly declining tail of the epidemic wave with some relatively mild oscillations. Black circles correspond to the data points. Different colors represent different sub-epidemics of the epidemic wave profile. The vertical line indicates the start time of the forecast

For the plague outbreak in Madagascar, the sub-epidemic model consistently outperformed the simpler models in short-term forecasts (2, 3, 4, and 5 weeks ahead) based on the MAE, the MIS, and the coverage of the 95% prediction interval (Table 3). In terms of the MSE, the sub-epidemic model outperformed the simpler models at forecasting horizons of 2 and 3 weeks, whereas the Richards model outperformed the other models at forecasting horizons of 4 and 5 weeks (Table 3, Figs. 8 and 9).

**Table 3:** Short-term forecasting performance in the context of the plague outbreak in Madagascar. Although the sub-epidemic model consistently outperformed the simpler models in terms of the quality of fit to the plague outbreak, the sub-epidemic model did not always outperform the Richards model based on all performance metrics in short-term forecasts. Values highlighted in italics correspond to the best performance metric at a given forecasting horizon

Model	Mean absolute error (MAE)	Mean squared error (MSE)	Mean interval score (MIS)	Percentage coverage of the 95% prediction interval
2 weeks ahead				
Sub-epidemic wave	*11.3*	*216.8*	*64.4*	*86*
Richards	13.2	275.0	101.3	76
Logistic	27.9	878.1	714.0	14
3 weeks ahead				
Sub-epidemic wave	*12.8*	*353.5*	*90.4*	*86.7*
Richards	14.9	392.0	112.0	74.7
Logistic	29.7	1003.0	792.0	12.0
4 weeks ahead				
Sub-epidemic wave	*14.4*	549.7	*115.9*	*85.0*
Richards	16.4	*508.0*	137.9	70.0
Logistic	31.3	1112.4	862.2	11.0
5 weeks ahead				
Sub-epidemic wave	*16.3*	878.5	*138.8*	*85.6*
Richards	17.5	*624.6*	164.6	65.6
Logistic	32.3	1197.0	919.1	10.4

**Fig. 10:**
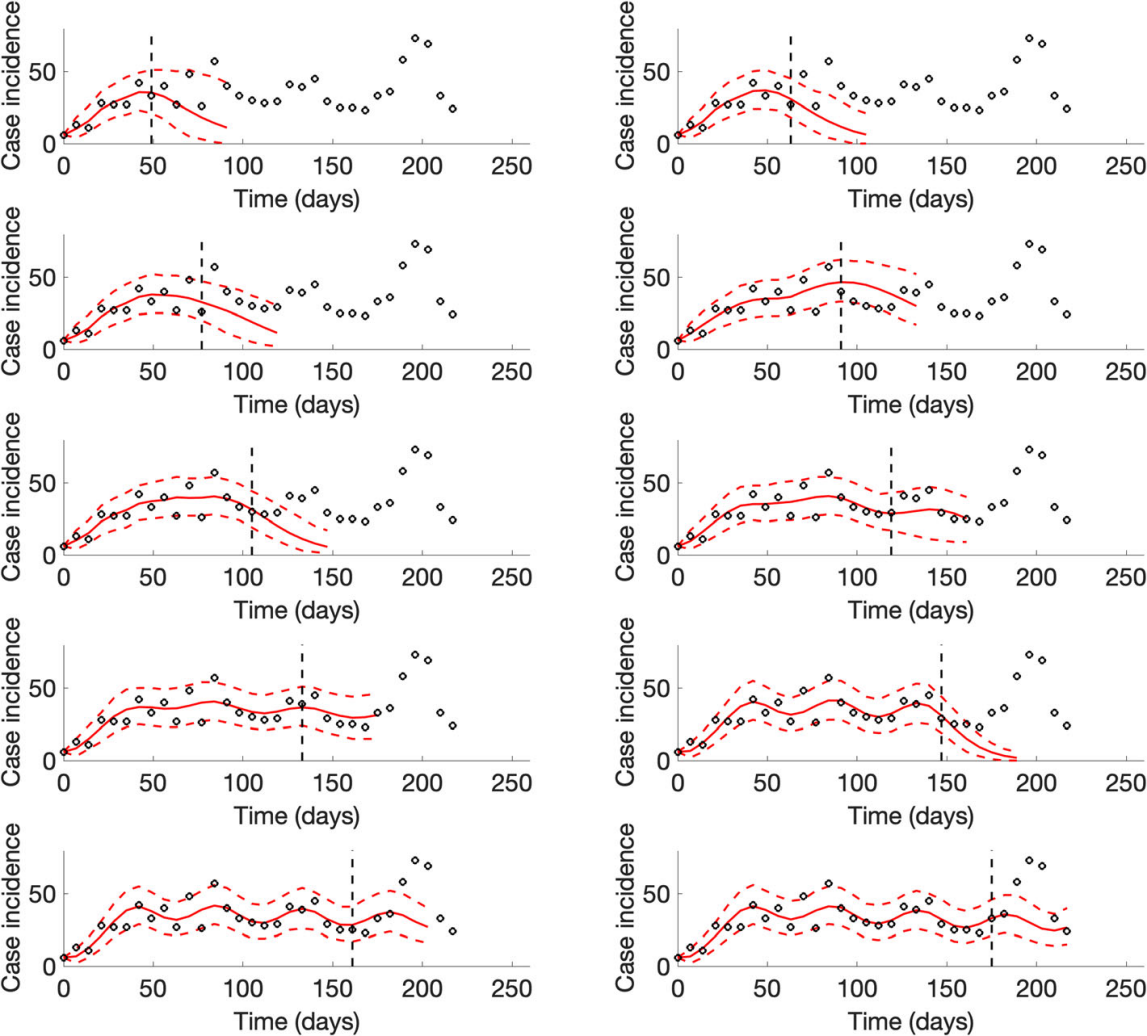
Representative 5-week ahead forecasts of the sub-epidemic model to the ongoing Ebola epidemic in DRC. Overall, we found that the epidemic wave model predicts a “traveling wave” with some oscillatory behavior that is shaped by a sub-epidemic profile of consecutive outbreaks. More specifically, the model consistently outperformed the simpler growth models in short-term forecasts based on all of the performance metrics (Table 4). However, the last forecast was unable to capture a significant increase in the incidence pattern associated with the fourth sub-epidemic of the epidemic wave profile shown in Fig. 11. Black circles correspond to the data points. The model fit (solid red line) and 95% prediction interval (dashed red lines) are also shown. The vertical line indicates the start time of the forecast

**Fig. 11:**
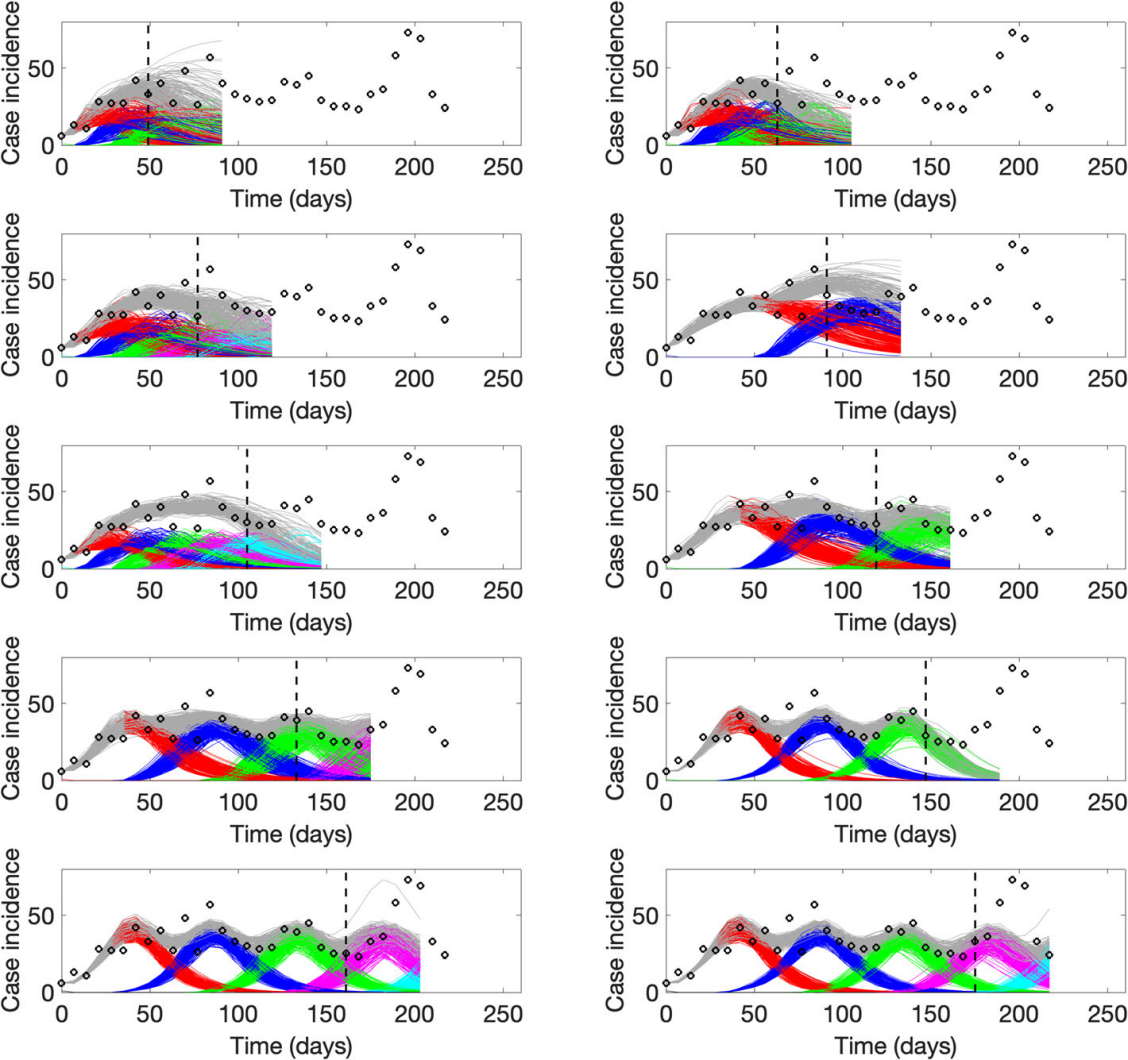
Sub-epidemic profiles of the epidemic wave forecasts for the Ebola epidemic in DRC displayed in Fig. 10. The sub-epidemic profiles of the forecasts derived for the ongoing Ebola outbreak in DRC exhibit consecutive and relatively stable sub-epidemics. Black circles correspond to the data points. Different colors represent different sub-epidemics of the epidemic wave profile. The vertical line indicates the start time of the forecast. The sub-epidemic model was unable to capture a significant increase in the incidence pattern associated with the fourth sub-epidemic of the epidemic wave profile

For the ongoing Ebola outbreak in DRC, the sub-epidemic model consistently outperformed the other models in short-term forecasts (2, 3, 4, and 5 weeks ahead) based on all of the performance metrics (Table 4). We found that the sub-epidemic model predicts a traveling wave with some oscillatory behavior that is shaped by a sub-epidemic profile of consecutive outbreaks (Fig. 10). However, the last forecast shows that the epidemic wave model was unable to capture a significant increase in the incidence pattern associated with a fourth sub-epidemic (Fig. 11).

**Table 4:** Short-term forecasting performance in the context of the Ebola outbreak in DRC. For the ongoing Ebola outbreak in DRC, the sub-epidemic model consistently outperformed the other models in short-term forecasts based on all of the performance metrics. Values highlighted in italics correspond to the best performance metric at a given forecasting horizon

Model	Mean absolute error (MAE)	Mean squared error (MSE)	Mean interval score (MIS)	Percentage coverage of the 95% prediction interval
2 weeks ahead				
Sub-epidemic wave	*9.3*	*131.9*	*67.4*	*86.1*
Richards	11.0	205.0	172.4	63.9
Logistic	21.3	555.2	538.5	13.9
3 weeks ahead				
Sub-epidemic wave	*10.9*	*194.7*	*104.8*	*79.6*
Richards	12.8	277.6	217.1	59.3
Logistic	23.8	689.0	658.4	9.26
4 weeks ahead				
Sub-epidemic wave	*12.3*	*258.5*	*153.1*	*75.0*
Richards	14.8	368.1	275.7	51.4
Logistic	26.0	828.9	768.9	7.0
5 weeks ahead				
Sub-epidemic wave	*14.1*	*337.9*	*207.1*	*68.9*
Richards	17.0	473.5	338.6	43.3
Logistic	28.1	975.4	874.3	5.6

## Discussion

We have introduced a sub-epidemic wave modeling framework based on the premise that overlapping and regular sub-epidemics, which are often unobserved, can determine the shape of the trajectory of epidemic waves that are observed at larger spatial scales. We demonstrated the framework by assessing the quality of model fit to observed case incidence data and performance in short-term forecasts for SARS in Singapore, plague in Madagascar, and the ongoing Ebola outbreak in DRC (September 2018 to mid-April 2019). We hope that our work will motivate the advancement of modeling frameworks and forecasting competitions that are needed for advancing the field of disease forecasting.

Our findings indicate that the sub-epidemic model outperformed simpler phenomenological growth models in short-term forecasts based on performance metrics that account for the uncertainty in predictions and was a better fit to epidemic trajectories from empirical outbreak data. The sub-epidemic modeling framework supports a diversity of epidemic growth dynamics including stable incidence patterns with sustained or damped oscillations. For example, the epidemic wave model successfully captured the bimodal pattern of the SARS outbreak in Singapore, and the short-term sequential model forecasts was able to forecast the second surge in case incidence for this outbreak. The second wave of the epidemic cannot be predicted by the simpler logistic growth models unless there is an external forcing term.

The sub-epidemic model for the Ebola epidemic in DRC indicates that the national incidence curve follows a stable incidence pattern with periodic behavior that can be decomposed into overlapping sub-epidemics. In particular, the epidemic wave model outperformed simpler phenomenological growth models in short-term forecasts of the Ebola epidemic in DRC. However, the model was unable to capture a significant recent increase in the incidence pattern, which highlights the need to strengthen public health interventions in the region in order to bring the epidemic under control. Such a significant increase in the incidence pattern could result from the deterioration in the effectiveness of contact tracing efforts and systematic vaccination refusals associated with community mistrust exacerbated by intermittent attacks to health workers and treatment centers.

Reporting delays tend to introduce substantial uncertainty in case incidence during the most recent weeks of an ongoing epidemic outbreak [35–37] and could potentially distort the incidence curve of the epidemic, and in turn, misconstrue estimates of transmission potential and forecasts of the outbreak trajectory. In the context of the ongoing Ebola epidemic in DRC [35], reporting delays are influenced by community mistrust in the government and public health authorities [40] as well as the effectiveness of control interventions (e.g., contact tracing and vaccinations) taking place in a conflict zone. Indeed, violent attacks continue to hamper the ongoing Ebola outbreak response, with recent attacks targeting Ebola treatment centers mainly located in Congo’s eastern areas of Butembo and Katwa [41, 42].

Our sub-epidemic modeling framework can capture a rich spectrum of epidemic dynamics compared to simple susceptible-infectious-removed (SIR) compartmental models which support early exponential growth in naïve populations and near symmetric epidemic trajectories [43, 44]. Our epidemic wave model supports traveling waves with oscillatory behavior with or without the effects of control interventions. Indeed, in large susceptible populations and in the absence of control interventions, traditional SIR compartmental models with homogenous mixing predict unabated exponential growth during the early epidemic growth phase in the absence of susceptible depletion and control interventions or behavior changes. Moreover, when calibrated with the trajectory of the initial growth phase, traditional models (e.g., logistic growth type models) tend to predict a near immediate decline in the epidemic trajectory [19] while our epidemic wave model forecasts traveling waves of variable shapes including resurgent epidemics stemming from the aggregation of asynchronous sub-epidemics [45]. Finally, post-peak forecasts using the epidemic wave model display an epidemic tail that tends to decline more slowly than predicted by traditional single-epidemic homogenous mixing SIR models [43].

Our findings highlight how overlapping sub-epidemic structures could give rise to oscillatory behavior or resurgence patterns in epidemic trajectories. This observation has significant implications for interpreting apparent noise in incidence data since the oscillations could be dismissed as the result of data overdispersion when in fact the oscillations stem from mechanisms underlying the transmission dynamics. More importantly, a real-time temporary downturn in case incidence resulting from oscillatory behavior could give the false impression of a positive effect of public health interventions.

Our epidemic wave model is phenomenological in the sense that multiple factors could be responsible for the predicted sub-epidemic profile ranging from the epidemiology of the disease to population mobility patterns, the distribution of risk behavior, and the effects of public health interventions. Prior studies have put forward relatively simple models that incorporate population structure and support traveling epidemic waves or disease resurgence patterns [45–51]). One such model is the household-community transmission model with overlapping communities that has been used to investigate transmission and control of Ebola epidemics [48]. In this model, outbreaks not only spread more slowly but the size of those epidemics is smaller compared to the homogenous mixing SIR models.

While the sub-epidemic wave model introduced in this paper is relatively simple, our work should motivate further development of more realistic multiscale models based on the sub-epidemic building block, perhaps by incorporating more complex dynamics for the generation of sub-epidemic profiles. For instance, in real-time epidemic forecasting applications, it could be possible to relax the assumptions regarding the regularity in the timing and evolution of sub-epidemic sizes in our model by relying on additional data stemming from field investigations. For instance, additional data could inform the timing and relative size of unfolding sub-epidemics. Furthermore, future work could investigate the forecasting performance of the sub-epidemic model with that of mechanistic models developed for specific diseases and contexts. Among mechanistic models, one could consider metapopulation transmission models that integrate sub-epidemics shaped by dynamic transmission rates or effective population sizes that fluctuate due to interventions or behavior changes.

## Additional file

Additional file 1: Incidence outbreak data for three real outbreaks. Daily number of new SARS cases by date of symptoms onset of the 2003 SARS outbreak in Singapore as explained in the text, weekly incidence curve according to the date of symptoms onset for the second wave of the ongoing Ebola outbreak in the DRC from the WHO Situation Reports and Disease Outbreak News covering the reporting period: September 2018 to mid-April 2019 as explained in the text, and weekly incidence curve of the 2017 plague epidemic in Madagascar as explained in the text. (XLS 32 kb)

## Data Availability

All of the data is shared in the supplement. Fitting code in MATLAB is available from: 10.6084/m9.figshare.8867882.v1
